# Effects of physical exercise on non-suicidal self-injury in adolescents: the chain mediating role of perceived social support and self-concept

**DOI:** 10.3389/fpsyt.2023.1201863

**Published:** 2023-10-09

**Authors:** Haoran Yu, Qinglei Mu, Ke Li

**Affiliations:** ^1^Institute of Sports Training, Chengdu Sport University, Chengdu, Sichuan, China; ^2^Institute of Physical Education, Chengdu University, Chengdu, Sichuan, China

**Keywords:** physical exercise, non-suicidal self-injury, perceived social support, self-concept, adolescents, intermediary role

## Abstract

**Objective:**

To study the effect of physical exercise on non-suicidal self-injury in adolescents and to verify the chain mediating role of perceived social support and self-concept. Methods: A survey study was conducted on 1,426 adolescents in Chengdu, Sichuan Province. A chain mediation model was used to verify whether perceived social support and self-concept played a mediating role.

**Results:**

Physical exercise was significantly negatively associated with non-suicidal self-injury in adolescents (β = −0.53, *p* < 0.01) and significantly positively associated with perceived social support and self-concept (β = 0.52, 0.54, *p* < 0.01), and perceived social support and self-concept were significantly negatively associated with non-suicidal self-injury (β = −0.59, *p* < 0.01; β = −0.64, p < 0.01), and perceived social support was able to significantly and positively associate self-concept (β = 0.76, *p* < 0.01).

**Conclusion:**

Perceived social support and self-concept play a chain mediating role in the effect of physical exercise on non-suicidal self-injury in adolescents, and it is recommended that the development of perceived social support and self-concept be emphasized during adolescents’ development, which has the potential to reduce the incidence of non-suicidal self-injurious behaviors in adolescents.

## 1. Introduction

In a report by the World Health Organization, it was stated that suicide is the second leading cause of death among young people aged 15–29 worldwide (1), where non-suicidal self-injury (NSSI) is an important predictor of suicidal behavior (2), and NSSI has become an increasingly serious global public health problem (3). Non-suicidal self-injury is defined as: an act in which an individual intentionally injures and directly damages his or her own body tissues without suicidal intent, commonly by cutting, stabbing, or excessive friction, which can cause bleeding and bruising, and this behavior is socially unacceptable (4, 5). In recent years, the prevalence of NSSI in the adolescent population and its harmful effects have received widespread attention, and a Meta-analysis showed that the percentage of Chinese secondary school students who had experienced NSSI behaviors was 22.37% (6). This indicates that Chinese adolescents are facing serious problems with the prevalence of NSSI behaviors and that individuals are less able to deal with their negative emotions at the adolescent stage and are prone to extreme NSSI behaviors (7). Given the high prevalence and negative effects of NSSI in adolescents, it is necessary to investigate and analyze the factors and mechanisms influencing NSSI.

A large number of studies at home and abroad have explored the risk and protective factors for suicidal behavior from a biopsychosocial as well as a cultural perspective, and physical exercise has been mentioned in some of these studies as a potential protective factor for suicidal behavior. Perceived social support is often used as a mediating variable, and participation in physical exercise promotes individuals’ access to social support, and perceived social support increases as a result. Perceived social support may serve as a protective factor for adolescents and may reduce mental health problems and high-risk behaviors (including non-suicidal self-injury). According to the multidimensional and hierarchical conceptualization of self-concept, physical exercise may be a contributing factor to improved self-concept, i.e., physical exercise may promote the development of self-concept. Non-suicidal self-injury fundamentally involves directing harmful behaviors toward the self; therefore, self-concept is a potential determinant of non-suicidal self-injury. Research suggests that more critical, hostile, or negative perceptions of oneself are associated with a greater risk of non-suicidal self-injury. The cognitive-emotional model of non-suicidal self-injury further emphasizes that self-concept is important in the occurrence of non-suicidal self-injury, i.e., self-concept may mitigate non-suicidal self-injury.

Physical exercise refers to physical activities in which people make their own choices according to their physical needs, use various means of physical activity and combine them with the forces of nature and hygienic measures in order to develop their bodies, improve their health, strengthen their physique, regulate their spirituality, enrich their cultural life and dispose of their leisure time (8). Numerous studies have shown that regular physical activity can reduce people’s stress, depression, and enhance their sense of well-being (9–11). Studies have shown that there is a strong link between mood and NSSI, with people with NSSI having higher levels of negative mood than those without NSSI (12). Emotion management theory states that when individuals are experiencing the effects of negative emotions, it is likely that NSSI will be used to manage negative emotions. NSSI is widely used as a means to reduce emotional distress when individuals have difficulty regulating negative emotions (13–15). Physical exercise has a significant negative impact on negative emotions, and participation in physical exercise can promote the secretion and release of β-endorphins in the body, making individuals feel pleasure and reducing the effects of negative emotions (16), which can reduce the frequency of NSSI. From a neurobiological perspective, physical exercise is considered to be one of the most direct and effective ways to alleviate negative emotions such as depression because of its ability to effectively regulate neurotrophic factor concentrations, glucocorticoids, and morphological structures in specific parts of the central nervous system (17). Numerous empirical studies have also proved that regular and moderate physical exercise has a good effect on reducing emotional experience, suppressing individual mood swings and serving to reduce the level of negative emotions (18). In addition, stressful life events (interpersonal relationships, family relationships, academic performance) are important influences on adolescents’ NSSI (19). Adolescents use NSSI as a way to regulate stress when experiencing stressful life events, and stressful life events increase the prevalence of NSSI in adolescents (20–22). There is a negative correlation between physical activity and stressful life events, and reasonable physical activity can enhance an individual’s frustration tolerance and thus reduce the negative impact of stressful life events (23). In addition, physical exercise can reduce negative psychological conditions and improve the psychological state of people with NSSI, thus reducing NSSI. Physical exercise may act as a protective mechanism, especially in individuals with depressive symptoms, to inhibit the behavioral impulses of NSSI (24). In summary, the following hypotheses were formulated in this study:

*Hypothesis 1 (H1)*: Physical exercise is negatively associated with non-suicidal self-injury in adolescents.

Perceived social support refers to the emotional experience and satisfaction of individuals who feel respected, supported and understood in society. It influences human behavior and development through the psychological reality of subjective perception of support, and exhibits a gainful function on individual mental health. Perceived social support is a part of social support, as opposed to actual social support (the actual help provided by elements of the surrounding environment when the individual is under stress or in distress), and is the individual’s perceptual evaluation of the social support received in the life environment, and the higher the level of perceived social support, the higher the level of life satisfaction (25). Research on social support has shown that perceived social support (subjective feelings) is more meaningful than objective social support and better highlights an individual’s level of mental health (26). Perceived social support may act as a protective factor against suicide and self-harm and reduce high-risk behaviors in adolescents (27, 28). When individuals have lower levels of perceived social support, they develop higher levels of negative emotions and have a higher probability of NSSI (29), and higher levels of perceived social support have a lower probability of NSSI in the face of stress or negative emotions (30), i.e., there is a negative correlation between perceived social support and NSSI. Social support theory suggests that one of the important ways to promote physical activity is to provide support before exercise, for example, by explaining how to exercise. In addition, physical exercise is not only an individual sport, but most of the time it needs to be done by multiple people, during which they are able to meet like-minded people according to their interests, promote social skills, and gain social support. Regular physical activity and increased interaction with family, friends, and others can promote the development of mental health and more social support perceived by individuals, i.e., there is a positive correlation between physical activity and perceived social support (31). In summary, the following hypotheses were formulated in this study:

*Hypothesis 2 (H2)*: There is a mediating role of perceived social support between physical activity and non-suicidal self-injury.

Self-concept refers to an individual’s knowledge and understanding of himself or herself, and is a person’s overall understanding of his or her own psychology, physical characteristics, values, behavior, and role positioning. Self-concept is an important foundation for an individual’s self-identity and self-esteem, as well as an important guide and determinant of an individual’s behavior and psychology (32). Studies have shown that physical activity is closely related to self-concept and that participation in physical activity can be effective in developing some elements of self-concept (33), i.e., physical activity can develop positive self-concept in adolescents and promote psychological health development by improving body satisfaction and body self-concept (34), and there is a positive correlation between physical activity and self-concept. It is clear from the concept of NSSI and the object of the behavior occurring that NSSI is fundamentally about inflicting hurtful behaviors on oneself, so the processes associated with perceiving the self may be important, and therefore self-concept is a potential determinant of NSSI (35, 36). Empirical studies have shown that adolescents with a history of NSSI have lower self-concept scores than their peers without a history of NSSI (37) and that adolescents with NSSI exhibit higher levels of worthlessness and insecurity compared to non-NSSI adolescents (38). The above findings further confirm that the association between self-concept and NSSI is significant (39). In summary, the following hypotheses were formulated in this study:

*Hypothesis 3 (H3)*: There is a mediating role of self-concept between physical activity and non-suicidal self-injury.

The above analysis suggested separate mediating roles for perceived social support and self-concept between physical activity and non-suicidal self-injury in adolescents. However, it has been shown that there is a correlation between perceived social support and self-concept, and that an increase in the level of perceived social support drives the development of self-concept; the more support individuals perceive from family, friends, and others, the higher their level of mental health, and thus the clearer their perception of self and the higher their level of self-concept, i.e., there is a positive correlation between perceived social support and self-concept (40, 41). Physical exercise promotes the development of individual perceived social support, adolescents feel more support from all aspects of society and improve their self-concept, and the combined effect of the two enhances adolescents’ positive state of mind and reduces the probability of NSSI. In summary, the following hypotheses are proposed in this study:

*Hypothesis 4 (H4)*: There is a chain mediating effect of perceived social support and self-concept between physical exercise and non-suicidal self-injury.

**Figure 1:**
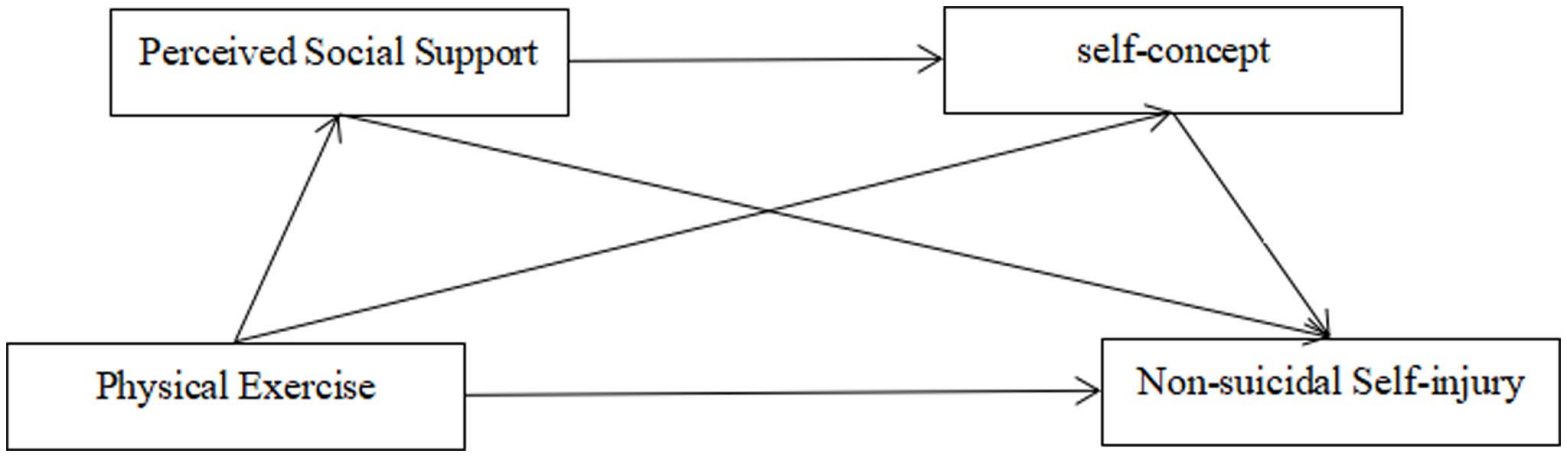
Hypothetical model diagram.

Based on the above hypotheses, a multiple mediation model of physical exercise and non-suicidal self-injury was constructed (Figure 1).

## 2. Methods

### 2.1. Participants and procedures

Based on the questionnaire-based research design, a random sampling method was used for students from 10 middle schools in Chengdu City, with up to 150 students from each middle school, students aged between 14 and 18 years old, and the questionnaires were sent to the participants between August and December 2022, with a total of 1,500 questionnaires distributed, and 1,426 valid questionnaires were received, with a sampling validity rate of 95.06%. The demographic characteristics of the sample, see Table 1. Inclusion criteria included (1) enrolled middle school students between the ages of 14 and 18 years old; (2) informed consent and voluntary participation; (3) ability to fill out surveys with the help of parents or the school microcomputer room; (4) having had at least one non-suicidal self-injurious behavior of varying degrees of severity. Exclusion criteria included (1) repeated invalid questionnaires and (2) questionnaires that took less than 1 min to complete. The study was conducted according to the guidelines of the Declaration of Helsinki and was approved by the Ethics Committee of Chengdu Sport University (code 2022–85, approved 15 August 2022). All tests were communicated to each participant before the survey began, and consent was obtained from the participants themselves, their schools, teachers, and parents.

**Table 1:** Demographic characteristics of the samples.

Variables		Frequency/Percentage (%)
Gender	Male	726 (50.91%)
	Female	700 (49.09%)
Family Location	Rural area	751 (52.66%)
	Urban area	675 (47.34%)
The Only Child in the Family	Yes	674 (47.27%)
	No	752 (52.73%)

### 2.2. Instruments

#### 2.2.1. Physical activity rating scale

The Physical Activity Rating Scale (PARS-3), revised by Liang Deqing, was used (42). The measurement contains 3 dimensions of physical exercise: time, frequency, and intensity. Both intensity and frequency were scored from 1 to 5, and time was scored from 0 to 4. The amount of physical exercise = intensity score × time score × frequency score. The assessment criteria were: ≤19 as small exercise; 20–42 as moderate exercise; ≥43 as large exercise. In this study, the Cronbach’s alpha coefficient was 0.62.

#### 2.2.2. Non-suicidal self-injury scale

The Adolescent Self-Injurious Behavior Questionnaire (43), developed by Feng Yu, was used to assess NSSI behaviors that occurred in adolescents during the past year. It was mainly used to assess the degree of NSSI behaviors of the subjects and included two subscales, self-injury frequency and self-injury degree. The frequency of self-injury was scored from 0 to 3, and the degree of self-injury was scored from 0 to 4. The total score = frequency of self-injury score × degree of self-injury score, with higher scores indicating higher levels of non-suicidal self-injury. In this study, the Cronbach’s alpha coefficient was 0.87.

#### 2.2.3. Perceived social support scale

The Chinese version of the Multidimensional Scale of Perceived Social Support (MSPSS) developed by Dahlem et al. (44) and translated and revised by Huang Li et al. (45) was used. The scale consists of 12 items, including three dimensions of individual perceived support from family, friends, and others, with four items each. The scores from “strongly disagree” to “fully agree” correspond to 1–7, respectively, and higher scores indicate higher perceived social support. In this study, the Cronbach’s alpha coefficient was 0.86.

#### 2.2.4. Self-concept scale

The Tennessee Self-Concept Scale (TSCT), developed by psychologist Fitts and revised by Lin Bang-Jie (46) in Taiwan, was used. The scale consists of 70 questions, including 2 dimensions of self-concept and composite status, with 10 factors to describe multidirectional self-concept. The higher the score on the first 9 factors, the more positive the self-concept, and the higher the score on the self-criticism, the more negative the self-concept. In this study, the Cronbach’s alpha coefficient was 0.93.

### 2.3. Statistical methods

Statistical analysis was performed using SPSS 26.0 and AMOS 24.0. This included common method bias test; independent sample *t*-difference test; Pearson correlation analysis; regression analysis of relevant variables; model fit analysis; and Bootstrap sampling test.

## 3. Results

### 3.1. Common method bias test

Since the data for the study came from a subjective questionnaire, a common method bias test was conducted on the variables involved in the study. Harman’s one-way test was used to test for common method bias, and 13 common factors with eigenvalues greater than 1 were obtained, and the first factor explained 27.32% (<40%), so the study did not have significant common method bias and met the statistical requirements (47).

### 3.2. Demographic differences

The demographic characteristics of the sample for this study included: gender, home location, and whether or not they were only children, and different gender, home location, and whether or not they were only children may differ on non-suicidal self-injury. Therefore, t-test and ANOVA were utilized to test whether different demographic characteristics in the sample differed on non-suicidal self-injury, see Table 2. There was no difference in non-suicidal self-injury among adolescents by gender (*p* = 0.400 > 0.05); no difference in non-suicidal self-injury by family location (*p* = 0.657 > 0.05); and no difference in non-suicidal self-injury among only children (*p* = 0.057 > 0.05).

**Table 2:** Analysis of variance in demographic variables.

Variables		Non-suicidal self-injury (Mean ± Standard deviation)
Gender	Male	1.53 ± 0.59
	Female	1.51 ± 0.60
	P	0.400
Family Location	Rural area	1.53 ± 0.59
	Urban area	1.51 ± 0.60
	P	0.657
The Only Child in the Family	Yes	1.55 ± 0.59
	No	1.49 ± 0.60
	P	0.057

### 3.3. Correlation analysis of each variable

Baron et al. showed that having correlation between the independent, dependent and mediating variables is a prerequisite for the analysis of mediating effects (48), and for this reason Pearson correlation analysis was performed for the four variables, see Table 3.

**Table 3:** Descriptive statistics and correlation of variables.

	*M*	SD	Physical exercise	Self-concept	Non-suicidal self-injury	Perceived family support
Physical Exercise	24.37	24.68	1			
Self-concept	2.72	0.65	0.54	1		
Non-suicidal Self-injury	1.52	0.59	−0.53	−0.64	1	
Perceived Social Support	2.72	0.72	0.52	0.76	−0.59	1

As shown in Table 3, physical exercise was significantly negatively correlated with non-suicidal self-injury (*r* = −0.53, *p* < 0.01), i.e., the level of non-suicidal self-injury may decrease with increased physical exercise. Physical exercise was significantly positively correlated with perceived social support (*r* = 0.52, *p* < 0.01) and self-concept (*r* = 0.54, *p* < 0.01), i.e., an increase in the amount of physical exercise may increase perceived social support and self-concept. Both perceived social support (*r* = −0.59, *p* < 0.01) and self-concept (*r* = −0.64, *p* < 0.01) were significantly negatively correlated with non-suicidal self-injury, and perceived social support was significantly positively correlated with self-concept (*r* = 0.76, *p* < 0.01). That is, increases in both perceived social support and self-concept may reduce the level of non-suicidal self-injury, and increases in perceived social support may promote the development of self-concept.

### 3.4. Regression analysis of each variable

As shown in Table 4, physical exercise was significantly and negatively associated with non-suicidal self-injury (β = −0.53, *p* < 0.01); therefore, Hypothesis 1 was supported; physical exercise was significantly and positively associated with perceived social support (β = 0.52, *p* < 0.01) and self-concept (β = 0.54, *p* < 0.01); both perceived social support and self-concept were significantly and negatively associated with non-suicidal self-injury (β = −0.59, *p* < 0.01; β = −0.64, *p* < 0.01); meanwhile, perceived social support was significantly positively associated with self-concept (β = 0.76, *p* < 0.01).

**Table 4:** Results of regression analysis of physical activity, perceived social support, self-concept, and non-suicidal self-injury.

	Non-suicidal self-injury			Self-concept			Perceived social support		
	*β*	*t*	*R* ^2^	*β*	*t*	*R* ^2^	*β*	*t*	*R* ^2^
Physical Exercise	−0.53	−23.51	0.28	0.54	24.27	0.30	0.52	22.87	0.27
Perceived Social Support	−0.59	−27.58	0.35	0.76	44.08	0.58			
Self-concept	−0.64	−31.29	0.41						

### 3.5. Intermediation effect test

**Figure 2:**
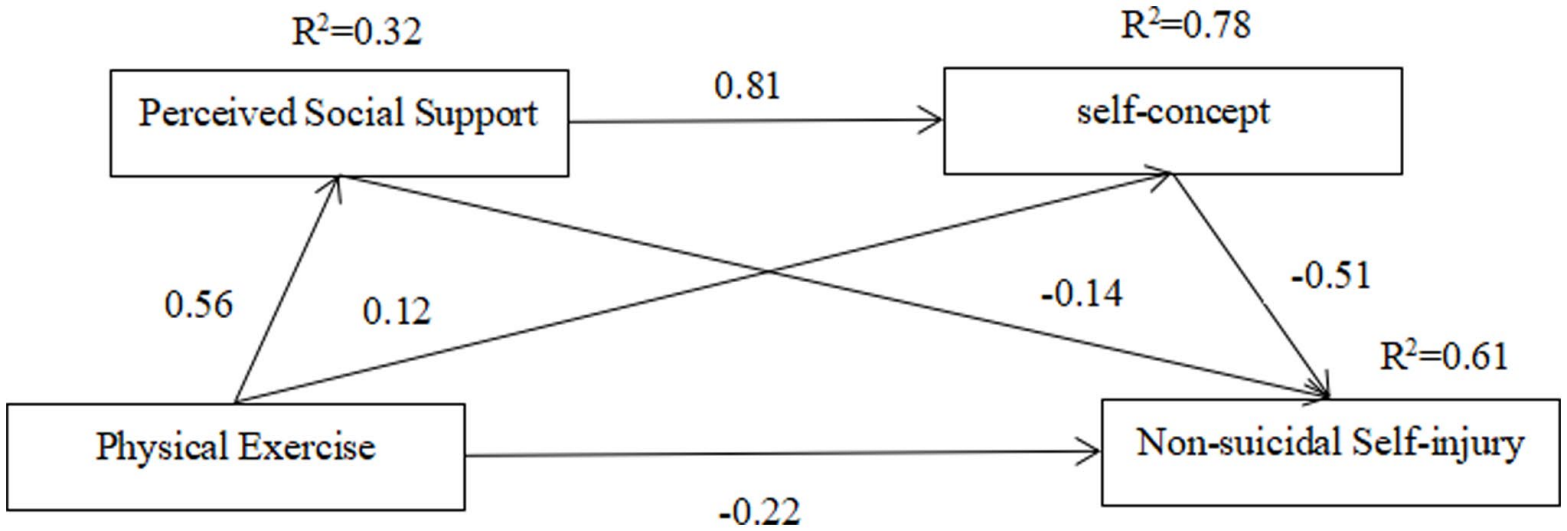
Structural equation model.

Wen et al. argued that it is more appropriate to use structural equation modeling to analyze the multiple mediation model (49). Based on this, AMOS 24.0 was used to establish structural equations for analysis, and the structural equation model diagram shown in Figure 2 was obtained. The existence of mediating effects and chain mediating effects of perceived social support and self-concept in the process of the influence of physical exercise on non-suicidal self-injury was examined.

The results of the goodness-of-fit test of the research model and data in this paper, see Table 5. Several key indicators meet the suggested values and reach the fit criteria, indicating that the overall goodness-of-fit of the theoretical model in this paper and the model diagram can be accepted, and the standardized path coefficients are shown in Figure 2.

**Table 5:** Fit index values of structural equation models.

	*χ* ^2^	*χ*^2^/*df*	CFI	NFI	RMSEA	RFI	IFI	AGFI
Indices	2140.59	1.31	0.98	0.92	0.02	0.92	0.98	0.95

As seen in Figure 2, physical exercise → non-suicidal self-injury (β = −0.22, *p* < 0.01), physical exercise → perceived social support (β = 0.56, *p* < 0.01), perceived social support → non-suicidal self-injury (β = −0.14, *p* < 0.01), physical exercise → self-concept (β = 0.12, *p* < 0.01), and self-concept → non-suicidal self-injury (β = −0.51, *p* < 0.01) all had significant path coefficients.

The mediation effect was verified using Bootstrap sampling test with 5,000 replicate samples. The mediation effect was verified using Bootstrap sampling test with 5,000 replicate samples. The results showed (Table 6) that perceived social support and self-concept played a fully mediating role between physical activity and non-suicidal self-injury, as shown in the following, physical activity → perceived social support → non-suicidal self-injury (95% CI: - 0.18 to −0.02), with a mediating effect size of −0.08; physical activity → self-concept → non-suicidal self-injury (95% CI: −0.10 ~ −0.03), with a mediated effect size of −0.06; and perceived social support → self-concept acted as a chain mediator, as evidenced by the fact that physical activity → perceived social support → self-concept → non-suicidal self-injury (95% CI, −0.33 to −0.15), with a mediated effect size of −0.23. The ratios of the three mediating effects to the total effect were 13.4, 10.7 and 39.3%, respectively. The 95% confidence interval of the three indirect effects did not contain the zero value, indicating that the three indirect effects were all significant. Therefore, hypotheses H2, H3, and H4 were supported.

**Table 6:** Analysis of intermediary effects.

	Effect	*SE*	LLCI	ULCI	Ratio to total effect
Physical Exercise→Non-suicidal Self-injury	−0.22	0.01	−0.28	−0.16	36.6%
Physical Exercise→Perceived Social Support→Non-suicidal Self-injury	−0.08	0.05	−0.18	−0.02	13.4%
Physical Exercise→Self-concept→Non-suicidal Self-injury	−0.06	0.02	−0.10	−0.03	10.7%
Physical Exercise→Perceived Social Support→Self-concept→Non-suicidal Self-injury	−0.23	0.04	−0.33	−0.15	39.3%

## 4. Discussion

### 4.1. Direct effect of physical exercise

The results of the study showed a negative association between physical exercise and non-suicidal self-injury in adolescents (β = −0.53, *p* < 0.01), with a direct effect of 36.6%. Hypothesis H1 was verified to be valid. The high prevalence and harm of NSSI in the adolescent population is mainly due to the fact that adolescents are prone to a number of psychological problems, such as: loneliness, interpersonal relationships, and depression (50, 51). Physical exercise has been proven to have a good effect on alleviating depressive symptoms, and teamwork during participation in sports allows adolescents to meet more friends with the same interests, reducing daily loneliness, drawing closer to peers through sports activities, and promoting interpersonal relationships. In-person sports activities can make adolescents feel supported and cared for by their parents, effectively controlling the generation of psychological problems and thus reducing the probability and frequency of NSSI (52–55). In summary, physical activity makes a positive contribution to the treatment and mitigation of NSSI in adolescents. Physical activity can be a potential treatment to reduce the probability of adolescent NSSI; for adolescents with NSSI, participation in physical activity may alleviate NSSI.

### 4.2. Mediating role of perceived social support

The results of this study showed that perceived social support had a separate mediating role between physical exercise and adolescent non-suicidal self-injury, with physical exercise positively associated with adolescent perceived social support (β = 0.518, *p* < 0.01) and perceived social support negatively associated with adolescent non-suicidal self-injury (β = −0.591, *p* < 0.01), with a mediating effect accounting for 13.4% of the total. Hypothesis H2 was verified to be valid. The effect of physical activity on adolescents’ perceived social support may be due to the fact that in team sports, adolescents gain the opportunity to interact with their peers, which helps them perceive support from their peers (56). When individuals encounter difficulties, care and help from family members will help them get through them more smoothly, and the more they perceive this outside support, the more they can actively solve problems and avoid the risk of NSSI occurring. When adolescents participate in physical activity and receive social support, they become more interested in physical activity, and conversely physical activity promotes the development of perceived social support. When adolescents perceive more social support, it also means more love and respect from various sources, such as family and friends. They will feel less rejection and refusal, and they will be in a relatively secure environment (57, 58). The development of perceived social support directly drives emotional de-escalation and reduces negative emotions, which in turn will reduce the likelihood of NSSI (59). In summary, perceived social support mediates the effect of physical activity on non-suicidal self-injury in adolescents and is negatively associated with adolescent NSSI. Adolescents’ participation in physical exercise may improve perceived social support, and perceived social support plays a positive role in alleviating adolescents’ non-suicidal self-injurious behavior. Improving adolescents’ ability to perceive social support through physical exercise may achieve the effect of alleviating non-suicidal self-injurious behavior and promote adolescents’ healthy development.

### 4.3. Mediating role of self-concept

This study found a separate mediating role for self-concept between physical activity and adolescent non-suicidal self-injury, with physical exercise positively associating with adolescent self-concept (β = 0.54, *p* < 0.01) and self-concept negatively associating with adolescent non-suicidal self-injury (β = −0.64, *p* < 0.01), with a mediating effect accounting for 10.7% of the total. Hypothesis H3 was verified to be valid. Physical exercise is not only a physical act, but there is also an interaction with the environment, and self-concept, as a psychological variable, is easily influenced by the external environment (60), and the physical satisfaction and psychological enjoyment that adolescents receive during physical exercise may be an important factor in promoting the development of self-concept, which is consistent with the results of previous studies (61). Adolescents’ NSSI is inflicted on themselves and is characterized by dissatisfaction with their bodies or a need to release stress, in short, a socially unacceptable maladaptive behavior (37). Self-concept is essentially a perception of the self, and when adolescents have a high level of self-concept, they have a clearer perception of the self and show more positive aspects, which reduces the probability of NSSI. In summary, self-concept plays a mediating role in the effect of physical exercise on non-suicidal self-injury in adolescents, physical activity has a positive effect on improving self-concept in adolescents, there is a negative correlation between self-concept and non-suicidal self-injury, and improved self-concept may reduce the probability of NSSI, and play a positive role in preventing and treating NSSI in adolescents.

### 4.4. Chain mediating role of self-concept and perceived social support

The results of this study indicated that perceived social support and self-concept were chained mediators between physical exercise and non-suicidal self-injury in adolescents, with perceived social support significantly and positively correlating with self-concept (β = 0.76, *p* < 0.01), with a mediating effect of 39.3%. Hypothesis H3 was verified to be valid. The chain mediating effect indicated that physical exercise enhanced adolescents’ perceived social support. The enhancement of perceived social support promoted the development of self-concept, which ultimately had a positive effect on improving adolescents’ NSSI. Perceived social support significantly and positively associated with self-concept, and the more support and care from family, friends and others, the higher the level of self-concept of individuals (41). When facing some difficulties in daily life and stresses that are difficult to relieve alone, support from all aspects of society can be used to get through them, while perceived social support and self-concept as very important psychological indicators for adolescents also play a positive role in the development of adolescent mental health, including reducing the probability of NSSI in adolescents.

### 4.5. Research limitations and perspectives

This study clarifies the relationship between physical exercise and non-suicidal self-injury and its underlying mechanisms, which has theoretical and practical implications. However, this study still has certain limitations and needs further improvement in the future. First, the physical exercise studied in this paper is referred to collectively as physical exercise in all sports, and its dimension needs to be extended so that future research can be conducted for specific sports items. Second, this study only considered the mediating role of perceived social support and self-concept, and it is unclear whether there are other effects between physical activity and non-suicidal self-injury in adolescents that can be further studied and analyzed in the future. Third, the data in this study came from subjective questionnaire tests of the subjects and lacked objective tests; objective tests could be added to the study to test the subjects in the future. Adolescents are a high prevalence group of non-suicidal self-injurious behavior and need more attention. Adolescents are a high prevalence group of non-suicidal self-injurious behaviors and need more attention. In the future, we should pay attention to the causes of non-suicidal self-injurious behaviors among adolescents; how to treat or prevent non-suicidal self-injurious injuries among adolescents; and how to alleviate non-suicidal self-injurious behaviors, and so on, in order to promote the healthy development of adolescents.

## 5. Conclusion

This study analyzed and validated the relationship between physical activity, perceived social support, self-concept, and non-suicidal self-injury in adolescents. It was found that gender, family location, and being an only child did not differ in terms of adolescent non-suicidal self-injury. Physical activity and adolescent non-suicidal self-injury are significantly negatively correlated and play an important role in reducing the probability of adolescent non-suicidal self-injury. Perceived social support and self-concept were chain-mediated and separately mediated between physical activity and adolescent non-suicidal self-injury. Participation in physical activity may promote the development of perceived social support and self-concept in adolescents, which contributes to the reduction in the probability of non-suicidal self-injury in adolescents.

Therefore, adolescents should participate in more physical exercise, which is conducive to healthy physical and mental development; society and families should give adolescents a variety of support to create a good environment for adolescents to grow up; adolescents should learn to pay more attention to their own development and change, and to improve various levels of cognition of the self; schools and societies should strengthen the dissemination of information on the hazards of non-suicidal self-injury and the prevention of such aspects, so that adolescents can scientifically understand non-suicidal self-injury, and consciously resist non-suicidal self-injury; conducting physical activity may be a potential solution to reducing the incidence of non-suicidal self-injurious behavior in adolescents, and physical activity could be included in treatment indicators when non-suicidal self-injurious behavior occurs in adolescents.

## Data availability statement

The raw data supporting the conclusions of this article will be made available by the authors, without undue reservation.

## Ethics statement

Written informed consent was obtained from the individual(s), and minor(s)' legal guardian/next of kin, for the publication of any potentially identifiable images or data included in this article.

## Author contributions

HY and QM: conceptualization, methodology, software, validation, formal analysis, writing—review and editing and resources. HY, QM, and KL: investigation and data curation. HY: writing—original draft preparation. All authors contributed to the article and approved the submitted version.

## References

[1] Li Y He K Xue C Li C Gu C. The impact of self-consistency congruence on non-suicidal self-injury in college students: the mediating role of negative emotion and the moderating role of gender. Int J Environ Res Public Health. (2022) 19:11898. doi: 10.3390/ijerph191911898, PMID: 36231200 PMC9564789

[2] Scott LN Pilkonis PA Hipwell AE Keenan K Stepp SD. Non-suicidal self-injury and suicidal ideation as predictors of suicide attempts in adolescent girls: a multi-wave prospective study. Compr Psychiatry. (2015) 58:1–10. doi: 10.1016/j.comppsych.2014.12.011, PMID: 25595520 PMC4369422

[3] Hasking P Rees CS Martin G Quigley J. What happens when you tell someone you self-injure? The effects of disclosing NSSI to adults and peers. BMC Public Health. (2015) 15:1039. doi: 10.1186/s12889-015-2383-0, PMID: 26453187 PMC4600263

[4] Ding H Zhu L Wei H Geng J Huang F Lei L. The relationship between cyber-ostracism and adolescents’ non-suicidal self-injury: mediating roles of depression and experiential avoidance. Int J Environ Res Public Health. (2022) 19:12236. doi: 10.3390/ijerph191912236, PMID: 36231539 PMC9564981

[5] Klonsky ED Olino TM. Identifying clinically distinct subgroups of self-injurers among young adults: a latent class analysis. J Consult Clin Psychol. (2008) 76:22–7. doi: 10.1037/0022-006X.76.1.22, PMID: 18229979

[6] Lang J Yao Y. Prevalence of nonsuicidal self-injury in Chinese middle school and high school students: a meta-analysis. Medicine. (2018) 97:e12916. doi: 10.1097/MD.0000000000012916 30335024 PMC6211880

[7] Yu L Ling X Jiang G. Impulsivity in non-suicidal self-injurious adolescents in China: impulsivity in non-suicidal self-injurious adolescents in China. Acta Psychol Sin. (2013) 45:320–35. doi: 10.3724/SP.J.1041.2013.00320

[8] Zhao Z Zhao S Wang Q Zhang Y Chen C. Effects of physical exercise on mobile phone addiction in college students: the chain mediation effect of psychological resilience and perceived stress. Int J Environ Res. Public Health. (2022) 19:15679. doi: 10.3390/ijerph192315679 36497752 PMC9738933

[9] Jayakody K Gunadasa S Hosker C. Exercise for anxiety disorders: systematic review. Br J Sports Med. (2014) 48:187–96. doi: 10.1136/bjsports-2012-091287 23299048

[10] Mailey EL Wójcicki TR Motl RW Hu L Strauser DR Collins KD. Internet-delivered physical activity intervention for college students with mental health disorders: a randomized pilot trial. Psychol Health Med. (2010) 15:646–59. doi: 10.1080/13548506.2010.498894, PMID: 21154018

[11] McGale N McArdle S Gaffney P. Exploring the effectiveness of an integrated exercise/CBT intervention for young men’s mental health: young men and mental health initiatives. Br J Health Psychol. (2011) 16:457–71. doi: 10.1348/135910710X522734, PMID: 21722270

[12] Victor SE Klonsky ED. Daily emotion in non-suicidal self-injury: daily emotion in NSSI. J Clin Psychol. (2014) 70:364–75. doi: 10.1002/jclp.22037 24002943

[13] Gratz KL. Targeting emotion dysregulation in the treatment of self-injury. J Clin Psychol. (2007) 63:1091–103. doi: 10.1002/jclp.20417 17932982

[14] Klonsky ED. The functions of deliberate self-injury: a review of the evidence. Clin Psychol Rev. (2007) 27:226–39. doi: 10.1016/j.cpr.2006.08.002, PMID: 17014942

[15] Nock MK Prinstein MJ. A functional approach to the assessment of self-mutilative behavior. J Consult Clin Psychol. (2004) 72:885–90. doi: 10.1037/0022-006X.72.5.885, PMID: 15482046

[16] Li C Jia H Zuo J. Effectiveness, mechanisms, and prospects of exercise for mental health. China Sport Sci Technol. (2015) 51:132–9. doi: 10.16470/j.csst.2015.01.016

[17] Wang M. Effects of adolescent exercise behavior on depressive tendencies: mediating effects based on motivation and subjective experience. Sports Sci. (2021) 42:78-85+110. doi: 10.13598/j.issn1004-4590.2021.06.012

[18] Yin J Xue Y. Moderate effects of exercise on negative affect under examination stress: a small sample of daily longitude study. J Tianjin Univ Sport. (2017) 32:443–7. doi: 10.13297/j.cnki.issn1005-0000.2017.05.011

[19] Gao Y Wang H Liu X Xiong Y Wei M. Associations between stressful life events, non-suicidal self-injury, and depressive symptoms among Chinese rural-to-urban children: a three-wave longitudinal study. Stress Health. (2020) 36:522–32. doi: 10.1002/smi.2954, PMID: 32369249

[20] Cipriano A Claes L Gandhi A Cella S Cotrufo P. Does anger expression mediate the relationship between parental rejection and direct and indirect forms of non-suicidal self-injury? J Child Fam Stud. (2020) 29:3575–85. doi: 10.1007/s10826-020-01844-9

[21] Steinhoff A Bechtiger L Ribeaud D Eisner M Shanahan L. Stressful life events in different social contexts are associated with self-injury from early adolescence to early adulthood. Front Psych. (2020) 11:487200. doi: 10.3389/fpsyt.2020.487200, PMID: 33192638 PMC7653177

[22] Tang J Yang W Ahmed NI Ma Y Liu H-Y Wang J-J. Stressful life events as a predictor for nonsuicidal self-injury in southern Chinese adolescence: a cross-sectional study. Medicine. (2016) 95:e2637. doi: 10.1097/MD.0000000000002637, PMID: 26945351 PMC4782835

[23] Shen X. Effects of exercise intervention and exercise-cognitive intervention on stress coping and psychological capacity of college students. J Wuhan Sports Univ. (2015) 49:76–82. doi: 10.15930/j.cnki.wtxb.2015.10.011

[24] Boone SD Brausch AM. Physical activity, exercise motivations, depression, and - self-injury in youth. Suicide Life Threat Behav. (2016) 46:625–33. doi: 10.1111/sltb.12240, PMID: 26970091

[25] Panayiotou G Karekla M. Perceived social support helps, but does not buffer the negative impact of anxiety disorders on quality of life and perceived stress. Soc Psychiatry Psychiatr Epidemiol. (2013) 48:283–94. doi: 10.1007/s00127-012-0533-6 22711064

[26] Hu J. (2022). School bullying and non-suicidal self-injury: the mediating role of negative affect and the regulation of perceived social support and its intervention.

[27] Kleiman EM Liu RT. Social support as a protective factor in suicide: findings from two nationally representative samples. J Affect Disord. (2013) 150:540–5. doi: 10.1016/j.jad.2013.01.033, PMID: 23466401 PMC3683363

[28] Lai CC Ma CM. The mediating role of social support in the relationship between psychological well-being and health-risk behaviors among Chinese university students. Health Psychol Open. (2016) 3:205510291667810. doi: 10.1177/2055102916678106, PMID: 28070409 PMC5193286

[29] Yamada Y Klugar M Ivanova K Oborna I. Psychological distress and academic self-perception among international medical students: the role of peer social support. BMC Med Educ. (2014) 14:256. doi: 10.1186/s12909-014-0256-3, PMID: 25430069 PMC4251849

[30] Geng Z Ogbolu Y Wang J Hinds PS Qian H Yuan C. Gauging the effects of self-efficacy, social support, and coping style on self-management behaviors in Chinese cancer survivors. Cancer Nurs. (2018) 41:E1–E10. doi: 10.1097/NCC.0000000000000571, PMID: 29461285

[31] Ren Y Li M. Influence of physical exercise on social anxiety of left-behind children in rural areas in China: the mediator and moderator role of perceived social support. J Affect Disord. (2020) 266:223–9. doi: 10.1016/j.jad.2020.01.152 32056881

[32] Simons J Capio CM Adriaenssens P Delbroek H Vandenbussche I. Self-concept and physical self-concept in psychiatric children and adolescents. Res Dev Disabil. (2012) 33:874–81. doi: 10.1016/j.ridd.2011.12.012 22245732

[33] Babic MJ Morgan PJ Plotnikoff RC Lonsdale C White RL Lubans DR. Physical activity and physical self-concept in youth: systematic review and meta-analysis. Sports Med. (2014) 44:1589–601. doi: 10.1007/s40279-014-0229-z, PMID: 25053012

[34] Fernández-Bustos JG Infantes-Paniagua Á Cuevas R Contreras OR. Effect of physical activity on self-concept: theoretical model on the mediation of body image and physical self-concept in adolescents. Front Psychol. (2019) 10:1537. doi: 10.3389/fpsyg.2019.01537, PMID: 31354570 PMC6635469

[35] Forrester RL Slater H Jomar K Mitzman S Taylor PJ. Self-esteem and non-suicidal self-injury in adulthood: a systematic review. J Affect Disord. (2017) 221:172–83. doi: 10.1016/j.jad.2017.06.027, PMID: 28647667

[36] Taylor PJ Dhingra K Dickson JM McDermott E. Psychological correlates of self-harm within gay, lesbian and bisexual UK university students. Arch Suicide Res. (2020) 24:41–56. doi: 10.1080/13811118.2018.1515136, PMID: 30152727

[37] Claes L Muehlenkamp J Vandereycken W Hamelinck L Martens H Claes S. Comparison of non-suicidal self-injurious behavior and suicide attempts in patients admitted to a psychiatric crisis unit. Personal Individ Differ. (2010) 48:83–7. doi: 10.1016/j.paid.2009.09.001

[38] Ross S Heath NL Toste JR. Non-suicidal self-injury and eating pathology in high school students. Am J Orthopsychiatry. (2009) 79:83–92. doi: 10.1037/a0014826, PMID: 19290728

[39] Andrews T Martin G Hasking P Page A. Predictors of onset for non-suicidal self-injury within a school-based sample of adolescents. Prev Sci. (2014) 15:850–9. doi: 10.1007/s11121-013-0412-8, PMID: 23812886

[40] Dorak F. The effect of a 12-week physical exercise program in adults on satisfaction with life, self-esteem, healthy lifestyle behaviors and perceived social support. Anthropologist. (2015) 19:469–77. doi: 10.1080/09720073.2015.11891681

[41] Xu Q Li S Yang L. Perceived social support and mental health for college students in mainland China: the mediating effects of self-concept. Psychol Health Med. (2019) 24:595–604. doi: 10.1080/13548506.2018.1549744, PMID: 30451537

[42] Liang D. Stress levels of students in higher education and their relationship with physical activity. Chinese J Mental Health. (1994) 5–6.

[43] Feng Y. (2008). The relation of adolecents’ self-harm behaviors, individual emotion characteristics and family environment factors. Available at: http://oversea.cnki.net.zzulib.vpn358.com/KCMS/detail/detail.aspx?dbcode=CMFD&dbname=CMFD2008&filename=2008116844.nh&uniplatform=OVERSEA&v=gj60OQSWvJHnYlYWgY5_RTbQBPzLw41SnTYTJDYFLYnIoW8a-F6WeKc5t6ga0Foy.

[44] Dahlem NW Zimet GD Walker RR. The multidimensional scale of perceived social support: a confirmation study. J Clin Psychol. (1991) 47:756–61. doi: 10.1002/1097-4679(199111)47:6<756:AID-JCLP2270470605>3.0.CO;2-L 1757578

[45] Huang L Jiang Q Ren W. Coping styles, social support and psychosomatic symptoms in cancer patients a study of the correlation between. Chin Ment Health J. (1996) 160–1.

[46] Tennessee Self-Concept Inventory (n.d.). Available at: http://sdtr.swu.edu.cn/s/sdtr/new13/20190108/3628809.html

[47] Zhou H Long L. Statistical remedies for common method biases. Adv Psychol Sci. (2004) 942–50.

[48] Baron RM Kenny DA. The moderator-mediator variable distinction in social psychological research: conceptual, strategic, and statistical considerations. J Pers Soc Psychol. (1986) 51:1173–82. doi: 10.1037//0022-3514.51.6.1173, PMID: 3806354

[49] Wen Z Ye B. Analyses of mediating effects: the development of methods and models. Adv Psychol Sci. (2014) 22:731–45. doi: 10.3724/SP.J.1042.2014.00731

[50] Luo Y Ren P Ma C Zhu J Wu R Liu Y. The mediating role of social support in the relationship between non_suicidal self-injury behavior and left-behind experien. Modern Prev Med. (2023) 50:535. doi: 10.20043/j.cnki.MPM.202208055

[51] Wang Z Jiang Z He Q Ma Y Yang J Cheng X. Correlation between behavioral inhibition/activation systems and adolescent non-suicidal self-injury: mediating effect of depression. Neurol Dis Mental Health. (2023) 23:25–31.

[52] Chen Z Yu P. The influence of physical exercise on subjective well-being of college students: an intermediary effect of peer relationship. J Cap Univ Phys Educ Sports. (2015) 27:165–71. doi: 10.14036/j.cnki.cn11-4513.2015.02.015

[53] Liu X Yang X. Physical activity intervention for autism spectrum disorders. Med Res Educ. (2020) 37:20–4.

[54] Zhao Y. Advances in antidepressant research on physical activity. J Guangzhou Sport Univ. (2014) 34:81–5. doi: 10.13830/j.cnki.cn44-1129/g8.2014.04.023

[55] Zhu Y Guo L Chen P Xu C. Peer relationship, sport motivation and behavior involvement among adolescents. J Tianjin Univ Sport. (2010) 25:218–23. doi: 10.13297/j.cnki.issn1005-0000.2010.03.010

[56] Jia N. Effects of physical exercise on social anxiety of rural left-behind children in Shaanxi Province and the mediating and moderating effects of perceived social support. Occup Health. (2021) 37:63. doi: 10.13329/j.cnki.zyyjk.2021.0014

[57] Turner BJ Cobb RJ Gratz KL Chapman AL. The role of interpersonal conflict and perceived social support in nonsuicidal self-injury in daily life. J Abnorm Psychol. (2016) 125:588–98. doi: 10.1037/abn0000141, PMID: 26845256 PMC5493473

[58] Yuan Z Li W Ding W Song S Qian L Xie R. Your support is my healing: the impact of perceived social support on adolescent NSSI — a sequential mediation analysis. Curr Psychol. (2023) 1–11:1–11. doi: 10.1007/s12144-023-04286-w

[59] Pejičić M Ristić M Anđelković V. The mediating effect of cognitive emotion regulation strategies in the relationship between perceived social support and resilience in postwar youth. J Community Psychol. (2018) 46:457–72. doi: 10.1002/jcop.21951

[60] Tian X Fu J. The impact of moderate to high intensity continuous exercise for junior middle school students’ self - concept and academic achievement. J Jilin Sport Univ. (2021) 37:50–5. doi: 10.13720/j.cnki.22-1286.2021.04.008

[61] Sun T Wang Z Yao J Ji C Dai Q Jin Y. Physical activity and mental health: advances in cognition, anxiety, depression, and self-concept. Prog Physiol Sci. (2014) 45:337–42. PMID: 25764792

